# MicroRNA suppression of stress-responsive NDRG2 during dexamethasone treatment in skeletal muscle cells

**DOI:** 10.1186/s12860-019-0194-3

**Published:** 2019-05-28

**Authors:** Bilal A. Mir, Rabia Islam, Ming Kalanon, Aaron P. Russell, Victoria C. Foletta

**Affiliations:** Institute for Physical Activity and Nutrition (IPAN), School of Exercise and Nutrition Science, Deakin University, Geelong, VIC 3222 Australia

**Keywords:** miRNA, NDRG2, Myotubes, Luciferase reporter assay, Stress response, Dexamethasone

## Abstract

**Background:**

MicroRNAs (miRNAs) are increasingly being identified as modulatory molecules for physiological and pathological processes in muscle. Here, we investigated whether miRNAs influenced the expression of the stress-responsive gene *N-myc downstream-regulated gene 2* (*Ndrg2*) in skeletal muscle cells through the targeted degradation or translation inhibition of NDRG2 mRNA transcripts during basal or catabolic stress conditions.

**Results:**

Three miRNAs, mmu-miR-23a-3p (miR-23a), mmu-miR-23b-3p (miR-23b) and mmu-miR-28-5p (miR-28), were identified using an in silico approach and confirmed to target the 3′ untranslated region of the mouse *Ndrg2* gene through luciferase reporter assays. However, miR-23a, -23b or -28 overexpression had no influence on NDRG2 mRNA or protein levels up to 48 h post treatment in mouse C2C12 myotubes under basal conditions. Interestingly, a compensatory decrease in the endogenous levels of the miRNAs in response to each other’s overexpression was measured. Furthermore, dexamethasone, a catabolic stress agent that induces NDRG2 expression, decreased miR-23a and miR-23b endogenous levels at 24 h post treatment suggesting an interplay between these miRNAs and NDRG2 regulation under similar stress conditions. Accordingly, when overexpressed simultaneously, miR-23a, -23b and -28 attenuated the dexamethasone-induced increase of NDRG2 protein translation but did not affect *Ndrg2* gene expression.

**Conclusion:**

These findings highlight modulatory and co-regulatory roles for miR-23a, -23b and -28 and their novel regulation of NDRG2 during stress conditions in muscle.

**Electronic supplementary material:**

The online version of this article (10.1186/s12860-019-0194-3) contains supplementary material, which is available to authorized users.

## Background

MicroRNAs (miRNAs) are small non-coding RNAs implicated as post-transcriptional regulators of fundamental skeletal muscle biological processes. Changes in their expression during muscle cell differentiation [1–3] and in response to stress and exercise [4–6] have resulted in the identification of miRNAs contributing to the control of muscle cell proliferation, tissue development, muscle regeneration and homeostasis [1, 3, 7–10]. MiRNAs may function through binding to specific regions of the 3′ untranslated region (3’UTR) of genes causing mRNA degradation or translational repression. For example, miR-23a binds to the 3’UTRs of fast myosin heavy isoforms contributing to the inhibition of myogenic differentiation [11]. MiR-23a also inhibits the translation of muscle-specific ubiquitin ligase genes, MAFbx/atrogin-1 and MuRF1, to protect against glucocorticoid dexamethasone-induced skeletal muscle atrophy [12]. Increased miR-181 expression enhances differentiation by suppressing homeobox protein HOX-A11, a MyoD negative regulator [3]. Other miRNAs and muscle-specific miRNAs (myomirs) are implicated in the control of skeletal muscle atrophy and hypertrophy processes [5, 13], myogenesis [14] and muscle-specific diseases (reviewed in [15]). While a number of miRNAs have been linked to the modulation of biological processes and stress responses in skeletal muscle, the genes they target to effect these changes are not well characterised.

N-myc downstream-regulated gene 2 (NDRG2) is a stress-responsive member of the NDRG protein family involved in preventing tumour growth and invasion (reviewed in [16]). MiR-650 directly targets NDRG2 and its upregulation is inversely associated with decreased NDRG2 expression in colorectal [17] and breast cancer cells [18]. Hypoxia-induced miR-301a/b targets the 3’UTR of *Ndrg2* resulting in NDRG2 protein suppression and increased autophagy and viability of prostate cancer cells [19, 20]. MiR-181c overexpression binds *Ndrg2’s* 3’UTR and downregulates its protein levels during cholangiocarcinogenesis and metastasis [21]. NDRG2 is also involved in a double-negative regulatory loop between leukemia inhibitory factor (LIF)/miR-181c where NDRG2 acts to inhibit LIF induction of miR-181c [21]. In adrenocortical carcinoma cells, miR-483-5p targets and suppresses NDRG2 to promote cancer invasion and pathogenesis [22]. Together, these studies highlight the interplay between miRNAs and NDRG2 function in cancer cells. There is currently very limited information regarding the regulation of NDRG2 by miRNAs in well-differentiated cell types such as skeletal muscle.

NDRG2 is well expressed in skeletal muscle [23] with expression increasing during muscle differentiation and development in vitro [24] and in vivo [25]. In muscle cells, NDRG2 promotes myoblast proliferation and protects against hydrogen peroxide-induced oxidative stress [26]. It is potentially associated with muscle mass changes where its expression is down and upregulated under anabolic and catabolic conditions, respectively, following dexamethasone treatment or resistance training [24]. The molecular factors regulating NDRG2 expression levels during myogenesis and in response to stress are poorly defined. While we identified the mouse *Ndrg2* gene as a target of the peroxisome proliferator-activated receptor-gamma coactivator-1alpha and estrogen-related receptor alpha transcriptional program [27], a role for miRNA regulation of NDRG2 in skeletal muscle cells is currently unknown.

In this study, we used miRNA prediction software and literature analysis to identify possible miRNAs that target the *Ndrg2* gene. Luciferase assays confirmed interactions of the predicted miRNAs with the mouse *Ndrg2* 3’UTR. The modulation of endogenous mRNA and protein levels of NDRG2 under basal and dexamethasone stress conditions following individual or combined miRNA overexpression was investigated in C2C12 myotubes.

## Materials and methods

### MicroRNA target prediction using in silico approaches

microRNA.org [28, 29] and miRWalk2.0 [30] softwares identified miRNAs predicted to target the 3’UTR region of mouse *Ndrg2* (NM_013864). To note, all known mouse *Ndrg2* variants have the same 3’UTR (https://www.ncbi.nlm.nih.gov/gene/29811). microRNA.org uses miRanda-predicted target sites with mirSVR scoring [28], and the miRWalk2.0 program enables the prediction of miRNA targets using a combination of the software programs: miRanda; miRWalk; RNA22; and Targetscan. MicroRNAs that were predicted both by microRNA.org and by all four software components of miRWalk2.0 were considered further. From these miRNAs, only those with a mirSVR score of − 0.7 to − 1.0 and an association with skeletal muscle biological processes in follow-up literature searches underwent further experimental validation.

### Dual luciferase reporter assay

The full length 868 bp 3’UTR fragment of the *Ndrg2* mRNA containing predicted miRNA binding sites was amplified by RT-PCR. The 3’UTR product was cloned downstream of the NanoLuc luciferase (*Nluc*) gene coding region in the pmirNanoGLO2 vector, which also contains the firefly luciferase (*luc2*) reporter gene for normalisation (Promega, Alexandria, NSW, AUS). The oligonucleotides designed to encompass the *Ndrg2* 3’UTR seed sequences for the predicted miRNA binding sites and their mutated equivalents are listed in Table 1. Approximately 1 × 10^5^ HEK293 cells (ATCC, Manassas, VA, USA) were plated in 96-well white-walled plates. The following day, 150 ng of each plasmid and 5 nM of each miRNA were co-transfected using Lipofectamine 2000 and Opti-MEM I reduced serum medium (Life Technologies, Mulgrave, VIC, AUS) as described by the manufacturer. Four hours post-transfection, the transfection mix was removed and replaced by growth medium containing 25 mM glucose Dulbecco’s Modified Eagle Medium (DMEM) with 10% fetal bovine serum. Twenty-four or 48 h later, cells were consecutively assayed for Nanoluc and Firefly luciferase expression using the Nano-Glo® Dual-luciferase® Reporter assay kit (Promega) following the manufacturer’s protocol. Normalized relative luciferase activity (RLA) was calculated as the following formula: RLA = [*Nluc*]/[*luc2* luciferase]. To note, C2C12 myoblasts were also assessed but HEK293 cells were consistently more reliable with their signal output and sensitivity than following transfection of the C2C12 myoblasts.

**Table 1:** Oligonucleotide sequences used for cloning of mouse *Ndrg2* 3’UTR, the predicted miRNA seed sites or their scrambled mutated versions

Oligonucleotide:	Sequence 5’-3’:
mNdrg2_3’UTR-F:	caccgctagcatgaccctcattgccttggtg
mNdrg2_3’UTR-R:	cgatctagaaacaatgctgttcagtttcctcta
miR23seed-F:	caccgctagcggtgggtcagtgatccttaatgtgatagaaatatccgcggg
miR23seed-R:	cgatctagaggatatttctatcacattaaggatcactgacccaccg
miR23mut-F:	caccgctagcggtgggtcagaatgttagtctcatgtagaaatatccgcggg
miR23mut-R:	cgatctagaggatatttctacatgagactaacattctgacccaccgagct
miR28seed-F:	caccgctagcttaacctgtgatatcctctagctcctaggtgaggccgcggg
miR28seed-R:	cgatctagaggcctcacctaggagctagaggatatcacaggttaagagct
miR28mut-F:	caccgctagcttaacctgttcatcgttactcactgcaggtgaggccgcggg
miR28mut-R:	cgatctagaggcctcacctgcagtgagtaacgatgaacaggttaagagct

### Cell culture and miRNA transfections

Mouse C2C12 myoblasts (ATCC) were cultured in growth medium at 37 °C in 5% CO_2_. To differentiate cells, medium consisting of 2% horse serum and 25 mM glucose DMEM was added when cells were confluent and refreshed every 48 h over a 6 day period. The miRNAs, mmu-miR-28-5p, mmu-miR-23a-3p, mmu-miR-23b-3p, mmu-miR-181a-5p or negative control (NC) miRNA (mirVana® miRNA mimics; Thermo Fisher Scientific, Scoresby, VIC, AUS) were transfected at 5 nM into differentiated myotubes using Lipofectamine 2000 and Opti-MEM I reduced serum medium (Life Technologies) as recommended. Myotubes were collected at 12, 24, or 48 h post transfection for NDRG2 mRNA and protein expression analyses. For mixed miRNA transfections, 5 nM of the three miRNAs were each combined together and transfected into myotubes with comparison to 15 nM NC miRNA transfected samples. For catabolic treatments, 10 μM dexamethasone (Sigma-Aldrich, Castle Hill, NSW, AUS) or 0.1% dimethyl sulfoxide (DMSO) vehicle were added to myotubes up to 48 h post-transfection and harvested concurrently.

### RNA extraction and gene expression analysis

Total RNA was isolated using Tri-Reagent (Ambion Inc., Austin, TX, USA), treated with DNAse I (Life Technologies), and quantitated using the NanoDrop 1000 spectrophotometer (Thermo Fisher Scientific). Half a microgram of RNA was reverse-transcribed to form cDNA using the High Capacity cDNA reverse transcription kit (Applied Biosystems, Foster City, CA, USA) according to manufacturer’s instructions. Semi-quantitative polymerase chain reaction (qPCR) was performed using the Mx3000 PCR system (Stratagene, La Jolla, CA, USA) with SYBR Green Master Mix (Applied Biosystems). The qPCR cycling conditions were: 95 °C for 10 min (1 cycle), 30 s at 95 °C and 60 °C for one min (40 cycles). Primers for mouse *Ndrg2* (NM_013864.2; forward 5’cccacacagacctcgttcc and reverse 5’gccatcgatggatgctgca) and a housekeeping gene, *36B4/Rplp0* (NM_007475.5; forward 5’ttgtgggagcagacaatgtg and reverse 5’agtcctccttggtgaacacg), were synthesized by GeneWorks (Adelaide, SA, AUS). To note, the primers for *Ndrg2* will detect all known variants of mouse *Ndrg2*. Samples were measured in duplicate using the Stratagene Mx3000P qPCR thermal cycler (Agilent Technologies, Mulgrave, VIC, AUS) and MxPRO qPCR software (Agilent Technologies). The relative gene expression is expressed as arbitrary units, which was calculated using the 2^−Δ*Ct*^ formula following normalization to *36B4* cycle threshold (Ct) levels.

### Western blotting

Cells were lysed in 1× modified RIPA buffer (50 mM Tris-HCl, pH 7.4, 150 mM NaCl, 0.25% deoxycholic acid, 1% NP-40, 1 mM EDTA) (Merck Millipore, North Ryde, NSW, AUS) containing dilution of 1:1000 protease inhibitor cocktail (Sigma-Aldrich) and 1:100 HALT phosphatase inhibitor cocktail (Thermo Fisher Scientific). Protein lysate concentrations were determined using Pierce™ BCA Protein Assay Kit (Thermo Fisher Scientific). Ten or twenty micrograms of protein lysates were electrophoresed on either 10% SDS-PAGE gels and transferred to Immobilon®-FL polyvinylidene difluoride membrane (Merck Millipore, Kilsyth, VIC, AUS). Membranes were blocked in 5% BSA for 1 h before incubation at 4 °C overnight in primary antibodies diluted in 5% BSA/PBS. Rabbit polyclonal anti-NDRG2 antibodies (HPA002896; 1:5000) were obtained from Sigma-Aldrich. Rabbit monoclonal anti-α-tubulin (clone DM1A; 1:5000) was obtained from Abcam (Cambridge, MA, USA). The detection of proteins was performed using goat anti-rabbit AlexaFluor®680 or donkey anti-mouse AlexaFluor®800 IgG antibodies (Life Technologies) diluted at 1:10,000 in 50% Odyssey blocking buffer (Li-COR Biosciences, Lincoln, NE, USA), 50% PBS and 0.01% SDS. Membranes were imaged using the infrared imaging system (Li-COR Biosciences). Proteins were normalized against α-tubulin protein levels using the Li-COR software.

### Detection of miRNA expression

MiRNA levels were measured by qPCR using specific primer and probes sets and the Taqman Universal MasterMix II, no UNG, kit as per the manufacturer’s instructions (Applied Biosystems, Carlsbad, CA). For miRNA expression analyses, total RNA (50 ng) was reverse transcribed using the Taqman microRNA Reverse Transcription (RT) kit (Applied Biosystems). A customized RT primer pool was prepared by pooling all miRNA-specific stem-loop primers. In brief, miRNA-specific primers (TaqMan® MicroRNA assay kits, Life Technologies) were pooled and diluted in nuclease free water to obtain a final dilution of 0.05x each in a volume of 15 μl containing 2 mM dNTP, 10 U enzyme, 1× RT buffer, 4 U RNase inhibitor and 50 ng of total RNA. The reaction mix was reverse transcribed using the following conditions: 30 min at 16 °C, 30 min at 42 °C and 5 min at 85 °C. The qPCR conditions consisted of 1 cycle of 10 min at 95 °C, and 40 cycles of 15 s at 95 °C and 60 s at 60 °C. The TaqMan™ microRNA snoRNA202 Control Assay was used as a normalizing control. Samples were run in triplicate for all miRNA targets and analysis was performed using the Stratagene MX3000P thermal cycler and dedicated software.

### Statistics

All data are reported as the mean ± standard error of the mean. Statistical differences were assessed using an unpaired Student’s t test for two-group comparisons at each time-point. For data involving three or more groups, data were subjected to one-way ANOVA with a Dunnett’s or Sidak’s multiple comparison test performed if a significant difference was found. Analyses were performed using GraphPad Prism (GraphPad Software version 7, La Jolla, CA, USA). Data were considered statistically significant if *p* < 0.05.

## Results

### miR-23a, -23b and -28 bind and inhibit the translation of *Ndrg2* 3’UTR

**Fig. 1:**
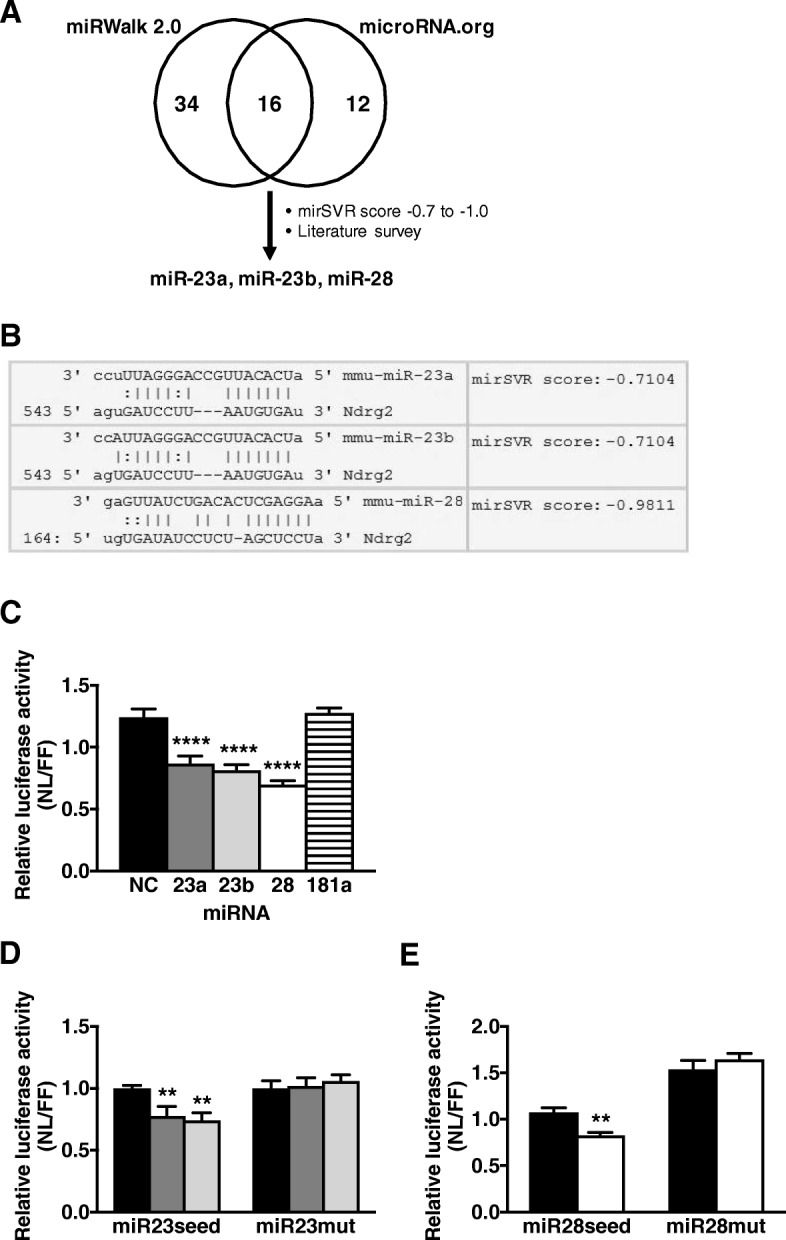
Selection and validation of predicted miRNAs binding to the mouse *Ndrg2* 3’UTR. **a** Venn diagram of miRNAs predicted from miRWalk2.0 and microRNA.org algorithms, and selection criteria used to identify potential miRNAs binding *Ndrg2* 3’UTR. **b** Nucleotide sequence alignment between the seed sites in the *Ndrg2* 3’UTR and predicted miRNAs with their corresponding mirSVR scores. **c** Dual-luciferase reporter assays determining the interaction of overexpressed miR-23a (dark grey bar), -23b (light grey bar), -28 (white bar), -181a (striped bar) and negative control (NC, black bar) with full-length *Ndrg2* 3’UTR; (**d**), with the miR-23a/−23b seed site (miR23seed) or its mutated version (miR23mut); and (**e**), with the miR-28 seed site (miR28seed) or its mutated version (miR28mut). Data is representative of three independent experiments with *n* = 5–6 per sample group, and expression levels are presented as arbitrary units (AU). **p < 0.01 and ****p < 0.0001 to NC

Using an in silico approach, 16 miRNAs were initially predicted by both microRNA.org and miRWalk2.0. Following further consideration of their mirSVR score [28] and whether they had reported associations with skeletal muscle biological processes in the literature, three miRNAs; mmu-miR-23a-3p (miR-23a), mmu-miR-23b-3p (miR-23b), and mmu-miR-28-5p (miR-28), remained as predicted to bind to the murine *Ndrg2* 3’UTR with both miR-23a and -23b binding to the same seed site (Fig. 1a-b). To confirm whether these miRNAs targeted *Ndrg2* 3’UTR in vitro, luciferase assays revealed miR-23a, -23b and -28 overexpression decreased *Ndrg2* 3’UTR-luciferase reporter activity by 30, 35 and 44%, respectively (Fig. 1c). To check the validity of our selection criteria, we also compared mmu-miR-181a-5p (miR-181a), which did not fit all the selection criteria. MiR-181a is associated with age-related muscle atrophy through binding to the *Sirt1* gene [31], and was predicted to bind to *Ndrg2* 3’UTR in microRNA.org (mirSVR score of − 1.0372). However, miR-181a was not identified using miRWalk2.0, and its overexpression had no impact on *Ndrg2* 3’UTR-luciferase reporter activity (Fig. 1c). To confirm the sites of interaction for the 3 miRNAs within the mouse *Ndrg2* 3’UTR region, luciferase assays were repeated using sequences containing the predicted seed site domains for miR-23a/‐23b and miR-28 binding. MiR-23a, ‐23b, and ‐28 overexpression each significantly reduced luciferase activity by 22, 26 and 17%, respectively, indicating an ability to bind to their respective seed sites. The reductions in luciferase activity were blocked when the miR-23a/‐23b or miR-28 seed sequences were mutated to prevent miRNA binding (Fig. 1d-e).

### MiRNA overexpression effects on endogenous NDRG2 mRNA and protein levels

**Fig. 2:**
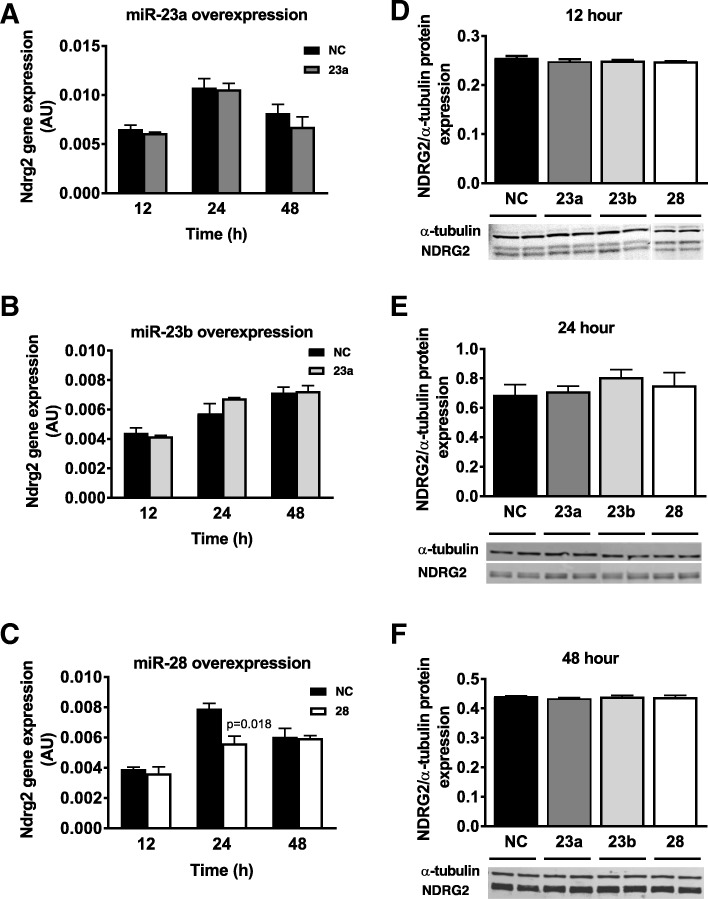
Impact of miRNA overexpression on endogenous mRNA and protein levels of NDRG2 under basal conditions. Time-course in hours (h) of *Ndrg2* mRNA expression following transfection of (**a**) miR-23a (dark grey bar), (**b**) miR-23b (light grey bar), and (**c**) miR-28 (white bar) compared to negative control (NC) miRNA (black bar). NDRG2 protein levels at (**d**) 12 h, (**e**) 24 h and (**f**) 48 h post miRNA transfection. Data are representative of two to three independent experiments with *n* = 3-4 per sample group, and expression levels are presented as arbitrary units (AU)

MiR-23a, miR-23b and miR-28 were overexpressed in C2C12 myotubes to evaluate if they could inhibit endogenous NDRG2 levels. Significant miRNA overexpression was achieved at each time point when compared to the respective negative control group (Additional file 1: Figure S1), which was included for each time point as it was noted that *Ndrg2* gene expression changed transiently in response to transfection and/or differentiation effects. No change in *Ndrg2* mRNA levels was measured at 12, 24 or 48 h following miR-23a or -23b overexpression (Fig. 2a-b); however, *Ndrg2* mRNA expression was reduced by 29% (*p* = 0.018) at 24 h post miR-28 transfection (Fig. 2c). Furthermore, no decrease in NDRG2 protein levels at any time point following transfection of either of the three miRNAs was identified (Fig. 2d-f).

### Regulation of endogenous miR-23a, -23b and -28 levels following miRNA overexpression or dexamethasone treatment in C2C12 myotubes

**Fig. 3:**
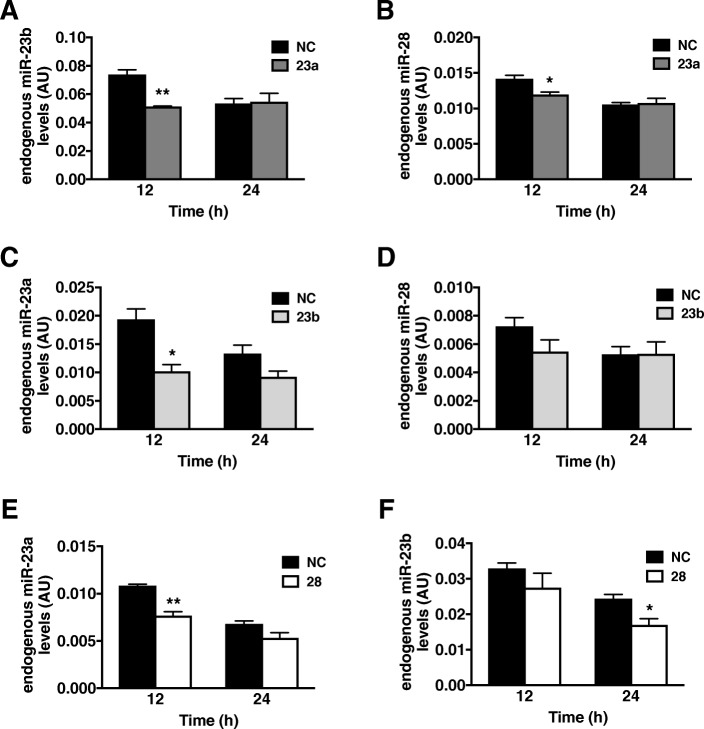
MiRNA overexpression downregulates endogenous miR-23a, -23b and -28 levels. Endogenous miRNA levels over time (h) following miRNA overexpression. **a** Endogenous miR-23b and (**b**) miR-28 levels following miR-23a overexpression (dark grey bar). **c** Endogenous miR-23a and (**d**) miR-28 levels following miR-23b overexpression (light grey bar). **e** Endogenous miR-23a and (**f**) miR-23b levels following miR-28 overexpression (white bar). Data are representative of two independent experiments with *n* = 4 per sample group; *p < 0.05 and **p < 0.01 to negative control (NC; black bar) per respective time point, and expression levels are presented as arbitrary units (AU)

As the overexpression of miR-23a, -23b and -28 did not reduce NDRG2 protein levels, we next investigated whether miRNA overexpression could potentially compensate for each other reducing their ability to inhibit NDRG2 expression. MiRNA cooperativity and redundancy in biological processes is evident in *C. elegans* worm studies [32, 33] and in cardiac muscle tissue where multiple miRNAs may be required to target a common gene in order to regulate a biological process (reviewed in [34]). Unexpectedly, we observed that miR-23a overexpression in myotubes resulted in the significant downregulation of endogenous miR-23a and -28 levels at 12 h post transfection (Fig. 3a-b). Similarly, miR-23b overexpression reduced endogenous miR-23a levels significantly at 12 h (Fig. 3c). Furthermore, miR-28 overexpression decreased endogenous miR-23a and miR-23b levels at 12 h and 24 h, respectively, post transfection (Fig. 3e-f).

**Fig. 4:**
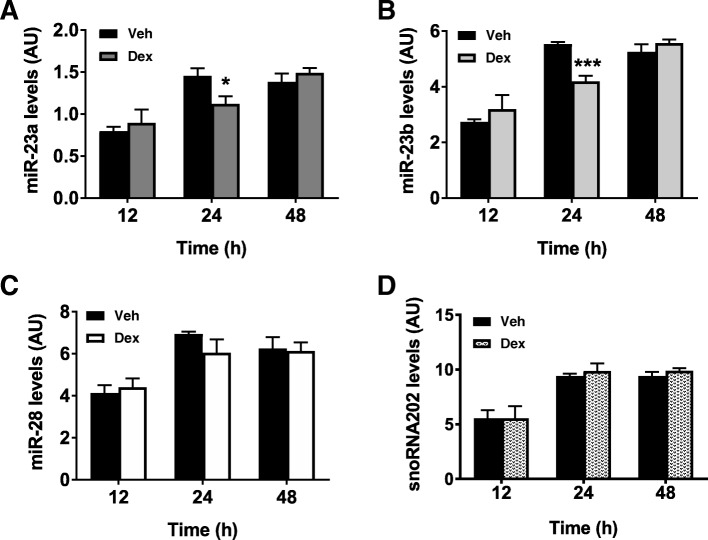
Endogenous miRNA levels following dexamethasone treatment. **a** Endogenous miR-23a, (**b**) miR-23b, (**c**) miR-28, and (**d**) snoRNA202 levels over time (h) following 10 μM dexamethasone (Dex) treatment. Data are representative of two independent experiments with n = 4 per sample group; *p < 0.05 and ***p < 0.001 to DMSO vehicle (Veh). Expression levels are presented as arbitrary units (AU)

A catabolic stress condition known to upregulate NDRG2 [24, 35] was next introduced. It has also been reported that this catabolic stress, the glucocorticoid dexamethasone, decreases miR-23a levels in C2C12 myotubes 48 h post treatment [36]. Here, we also observed a downregulation of miR-23a, but at 24 h, not 48 h post dexamethasone treatment (*p* < 0.05; Fig. 4a). In addition, miR-23b expression levels were decreased 24 h post treatment which was not sustained at 48 h (*p* < 0.001; Fig. 4b). No significant regulation by dexamethasone of miR-28 (Fig. 4c) or of the miRNA control gene, snoRNA202, was measured (Fig. 4d).

### Combined overexpression of miR-23a, -23b and -28 blocks NDRG2 induction during catabolic stress conditions

**Fig. 5:**
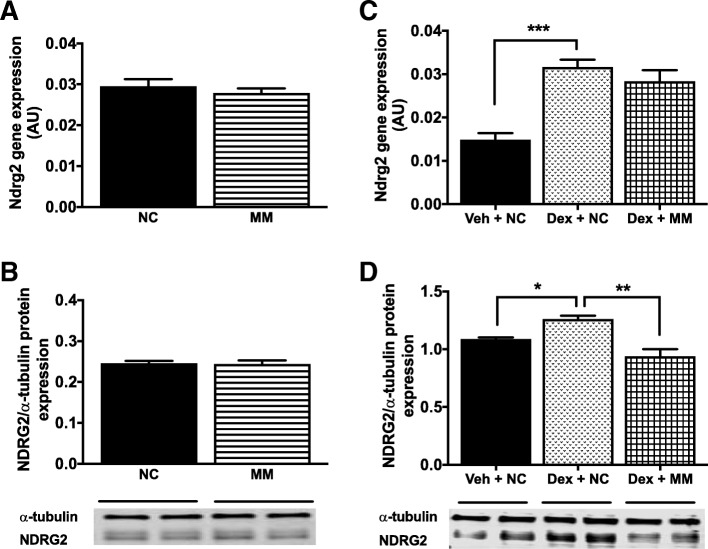
The effect of combined miRNA overexpression on endogenous NDRG2 mRNA and protein levels under basal and catabolic conditions. miR-23a, -23b and -28 were co-transfected into myotubes and their combined effects on NDRG2 regulation was measured in the presence or absence of 48 h treatment with 10 μM dexamethasone. **a** *Ndrg2* mRNA and (**b**) NDRG2 protein levels under basal conditions following miRNA co-treatment (striped bar) or negative control miRNA (NC, black bar) transfection. **c** *Ndrg2* mRNA and (**d**) NDRG2 protein levels following the combined effects of DMSO vehicle and negative control miRNA treatment (Veh + NC; grey bar), dexamethasone and negative control miRNA treatment (Dex + NC; stippled bar), or dexamethasone and miRNA mix treatment (Dex + MM; cross-hatched bar). Data are representative of two independent experiments with n = 4 per sample group, and expression levels are presented as arbitrary units (AU). *p < 0.05, **p < 0.01 or ***p < 0.001 to Dex + NC treatment

As endogenous miR-23a, -23b and -28 levels were reduced by the overexpression of each other, and miR-23a and -23b decreased with dexamethasone treatment, these findings suggest an interplay between the three miRNAs and also with *Ndrg2*’s stress-response regulation by dexamethasone [24, 35]. Therefore, a cocktail mix of the three miRNAs was transfected into myotubes to determine if their combined overexpression could regulate NDRG2 levels in the presence or absence of dexamethasone. Transfection of the miRNA cocktail mix achieved significant overexpression of miR-23a, -23b and -28 simultaneously in myotubes (Additional file 2: Figure S2); however, no change was observed in *Ndrg2* mRNA or NDRG2 protein levels under basal conditions (Fig. 5a-b). In the presence of dexamethasone at 48 h post treatment, *Ndrg2* mRNA and NDRG2 protein levels increased by approximately 100 and 20%, respectively, in C2C12 myotubes (Fig. 5c-d); however, the induction of NDRG2 protein by dexamethasone was blocked following the combined overexpression of the three miRNAs (*p* = 0.0012; Fig. 5d). No impact on *Ndrg2* mRNA expression under these same conditions was measured (Fig. 5c).

## Discussion

The aim of this study was to identify a novel mechanism of NDRG2 regulation in skeletal muscle cells with a specific focus on miRNAs. Using an in silico approach, miR-23a, -23b and -28 were identified and their potential interaction with mouse *Ndrg2* 3’UTR confirmed using cell-based biochemical assays. Individual or combined miRNA overexpression did not inhibit endogenous NDRG2 under basal conditions. However, the combined overexpression of the miRNAs inhibited the induction of NDRG2 protein expression following dexamethasone treatment. These results demonstrate a novel interplay between miRNAs and the regulation of NDRG2 under catabolic conditions in myotubes.

The importance of miRNAs in skeletal muscle development and homeostasis is highlighted in mice expressing a skeletal muscle-specific deletion of Dicer, an endonuclease required for miRNA processing. Mice unable to process miRNAs within skeletal muscle develop muscle hypoplasia, reduced muscle mass and perinatal death [37]. MiRNAs are also implicated in the control of muscle regeneration, dystrophies, myopathies, and in the development of cardiovascular and neurodegenerative diseases (reviewed in [15, 34]). How miRNAs function in muscle tissues is complex as they are reported to potentially ‘buffer’ against physiological and pathological changes to maintain homeostasis. To do this, they may target multiple gene transcripts in the same pathway to ensure a biological outcome, or display cooperativity and redundancy by synergistically targeting the same transcript [15, 34]. The latter scenario may be the case in our study where the overexpression of miR-23a, -23b and -28 caused the endogenous downregulation of the other miRNAs that suggests a level of redundancy and cooperativity in their regulation and function. Furthermore, the combined overexpression of the three miRNAs in the presence of dexamethasone prevented the increase in NDRG2 translation. This outcome maybe a reflection of the modulatory regulation of NDRG2 in stress scenarios but also emphasises the roles miRNAs play in disease and stress conditions experienced in skeletal muscle tissue. For example, miR-206-deficient mice display normal neuromuscular junction (NMJ) formation in healthy skeletal muscle although following the introduction of stress such as an acute injury causing muscle regeneration, new NMJ formation was impaired [9]. Furthermore, miR-206’s deficiency exacerbated disease progression in a mouse model of motor neurone disease [9] . Whether all three miRNAs are required to act cooperatively or can function independently to inhibit NDRG2 increase during dexamethasone and other catabolic stress are yet unknown. However, since the effect of dexamethasone on NDRG2 levels is moderate, it is unlikely that individually, miR-23a, -23b and -28 will have a substantial effect on NDRG2 regulation. The potential co-regulation of NDRG2 by multiple miRNAs in skeletal muscle is further supported by a similar scenario reported recently in the lumbar region of embryonic sheep where target genes, including *NDRG2,* are believed co-regulated by multiple miRNAs [38].

Each miRNA predicted to inhibit *Ndrg2* in this study plays roles in skeletal muscle biological processes. miR-23a, -23b and -28 are all linked to myogenesis and differentiation [11, 39–41] with miR-23a also associated with muscle wasting [36, 42, 43]. Previously, miR-23a was reported to decrease with dexamethasone in C2C12 myotubes at 48 h post treatment [36], and in miR-23a transgenic mice, miR-23a reduced dexamethasone-induced muscle wasting potentially through the translational inhibition of the ubiquitin proteasomal E3 ligases, MAFbx/atrogin-1 and MuRF1 [12]. Currently, these two studies do not delineate a clear role for miR-23a in dexamethasone induced-stress as its downregulation is counter-intuitive to a function involving MAFbx and MuRF1 inhibition. Here, we observed the downregulation of both miR-23a and miR-23b by dexamethasone, albeit at 24 h, not 48 h post treatment. Previous studies have demonstrated that dexamethasone treatment increases *Ndrg2* transcription in murine skeletal muscle cells [24] and in rat astrocytes [35, 44] with the induction of *Ndrg2* by dexamethasone occurring indirectly through nuclear factor-κB or paired box gene 5 transcription factor binding [35]. Given our findings here, it is tempting to speculate that the decrease in miR-23a and -23b endogenous levels by dexamethasone may counteract their ability to block NDRG2’s stress response increase to dexamethasone. Regardless, these findings suggest a novel regulatory mechanism of NDRG2 translation by miRNAs during stress conditions. Furthermore, it appears that these miRNAs function through their binding to the 3’UTR of *Ndrg2,* rather than degradation of *Ndrg2* transcripts, two known mechanisms by which miRNAs can function to control gene and protein expression (reviewed in [45]).

While miR-23a and -23b have not been described elsewhere to target human or rodent *Ndrg2*, miR-28-5p was reported recently to target and suppress human *NDRG2* in chronic lymphocytic leukemia cells [46]. This miRNA regulation was proposed as a mechanism to help suppress *NDRG2* expression in cancer cells along with epigenetic silencing. This study used in silico and similar in vitro validation approaches involving dual luciferase assays in HEK293T where the authors identified miR-28 to bind to a predicted target sequence in the 3’UTR region of human *NDRG2* [46], which helps validate our findings reported here of mouse *Ndrg2* regulation by miR-28-5p.

## Conclusion

Here, novel co-regulation of mouse *Ndrg2* by miRNAs under specific stress conditions in skeletal muscle cells is described. However, the in vitro overexpression of the miRNAs in the presence of dexamethasone represents non-physiological conditions for the regulation of *Ndrg2*. Whether miR-23a, -23b and -28 act cooperatively under similar stress conditions in vivo, and whether *Ndrg2* represents a novel therapeutic target in catabolic muscle wasting conditions remains to be determined.

## Additional files

Additional file 1: **Figure S1.** miR-23a, -23b and -28 expression levels following miRNA transfection in C2C12 myotubes. Time-course in hours (h) of miR-23a, -23b and -28 expression levels following transfection of (A) miR-23a (dark grey bars), (B) miR-23b (light grey bars), and (C) miR-28 (white bars) compared to negative control (NC) miRNA (black bars) at 12, 24 or 48 h post transfection. Data are representative of two independent experiments with *n* = 4 per sample group. **p < 0.01 and ***p < 0.001 to each NC per respective time point, and expression levels are presented as arbitrary units (AU). (DOCX 37 kb)

Additional file 2: **Figure S2.** MiRNA expression following combined miRNA mix transfection. MiRNA levels of (A) miR-23a (dark grey bars), (B) miR-23b, (light grey bars), and (C) miR-28 (white bars) following transfection of miRNA mix or negative control (NC) miRNA (black bars) at 48 h post transfection. Data are representative of two independent experiments with n = 4 per sample group. ***p < 0.001 to NC, and expression levels are presented as arbitrary units (AU). (DOCX 101 kb)

## Abbreviations

3’UTR: 3’ untranslated region
AU: arbitrary units
miRNA: microRNA
NC: negative control
NDRG2: N-myc downstream-regulated gene 2
