# Supplementary material for: MicroRNA suppression of stress-responsive NDRG2 during dexamethasone treatment in skeletal muscle cells
Source: BMC Mol Cell Biol. 2019 May 28;20:12. doi: 10.1186/s12860-019-0194-3 (PMC6537443; doi:10.1186/s12860-019-0194-3)
Supplement: Supplementary file 1 [file 12860_2019_194_MOESM1_ESM.docx]

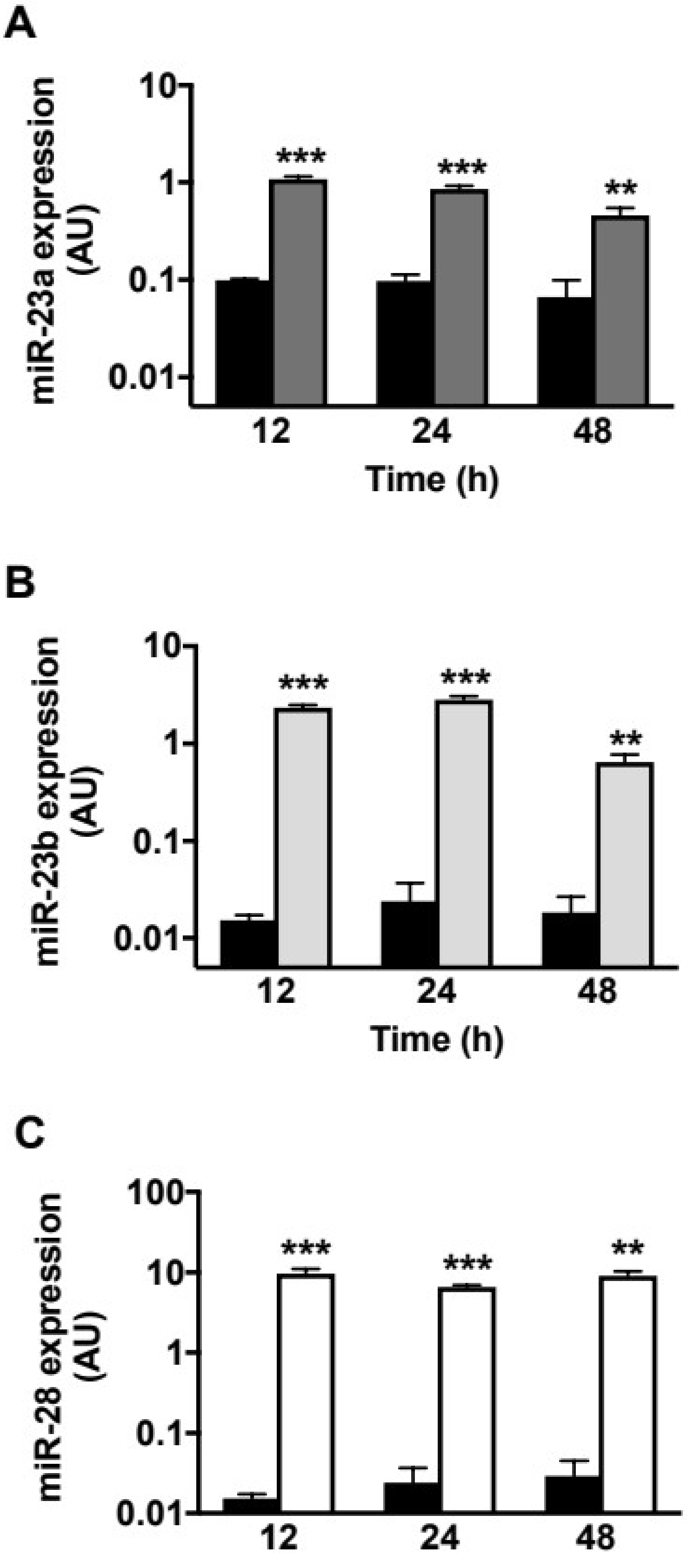
A
miR-23a expression (AU)
10
1
0.1
0.01
***
***
**
12
24
48
Time (h)
B
miR-23b expression (AU)
10
1
0.1
0.01
***
***
**
12
24
48
Time (h)
C
miR-28 expression (AU)
100
10
1
0.1
0.01
***
***
**
12
24
48
Time (h)
