# Supplementary material for: MicroRNA suppression of stress-responsive NDRG2 during dexamethasone treatment in skeletal muscle cells
Source: BMC Mol Cell Biol. 2019 May 28;20:12. doi: 10.1186/s12860-019-0194-3 (PMC6537443; doi:10.1186/s12860-019-0194-3)
Supplement: Supplementary file 2 [file 12860_2019_194_MOESM2_ESM.docx]

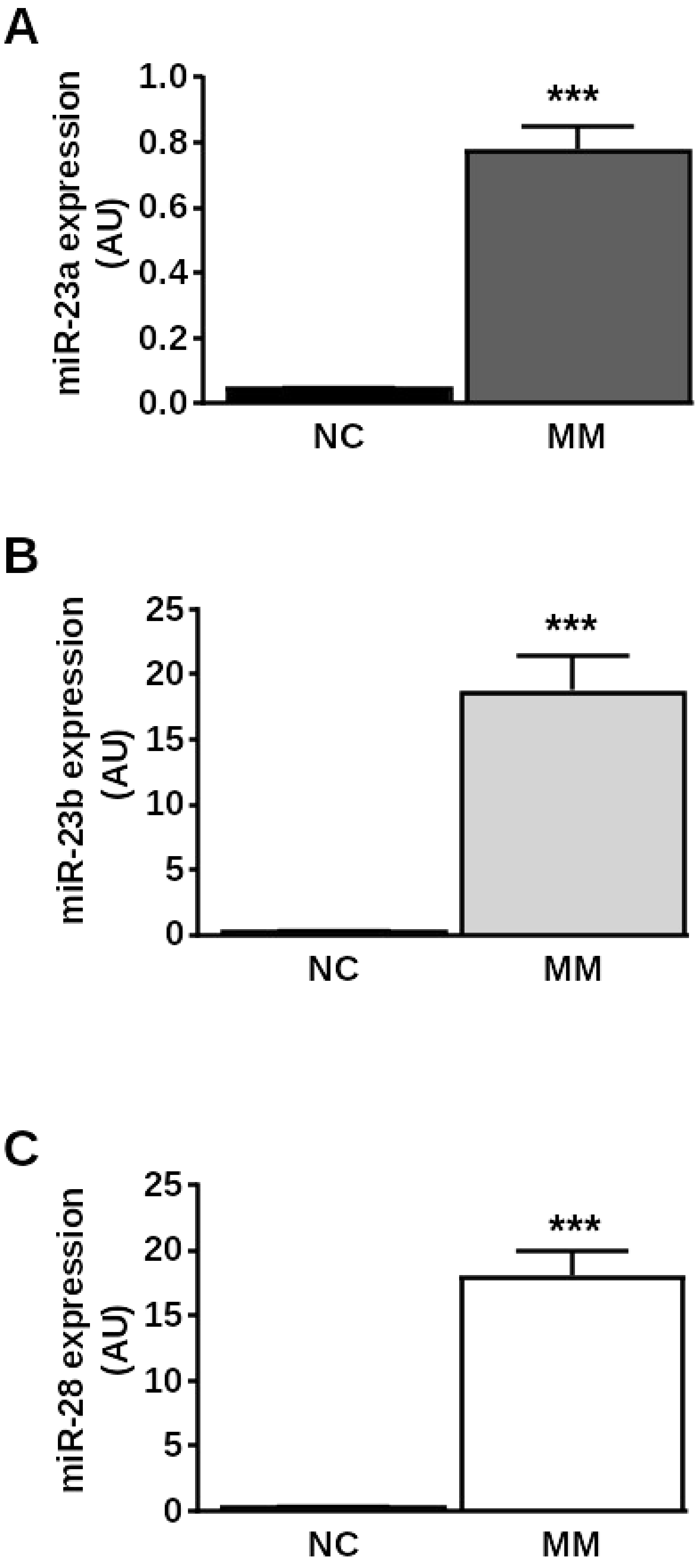
A
miR-23a expression (AU)
1.0
0.8
0.6
0.4
0.2
0.0
***
NC
MM
B
miR-23b expression (AU)
25
20
15
10
5
0
***
NC
MM
C
miR-28 expression (AU)
25
20
15
10
5
0
***
NC
MM
